# Predictors of overweight and obesity in adult women in Nairobi Province, Kenya

**DOI:** 10.1186/1471-2458-12-823

**Published:** 2012-09-25

**Authors:** Regina W Mbochi, Elizabeth Kuria, Judith Kimiywe, Sophie Ochola, Nelia P Steyn

**Affiliations:** 1 Department of Foods, Nutrition and Dietetics, Kenyatta University, Nairobi, Kenya; 2 Centre for the Study of Social and Environmental Determinants of Nutrition; Population Health, Health Systems, and Innovation, Human Sciences Research Council, P Bag X9182, Cape Town, 8000, South Africa

**Keywords:** Overweight, Obesity, Women, Kenya, Diet, Physical activity

## Abstract

**Background:**

Since obesity in urban women is prevalent in Kenya the study aimed to determine predictors of overweight and obesity in urban Kenyan women.

**Methods:**

A cross-sectional study was undertaken in Nairobi Province. The province was purposively selected because it has the highest prevalence of overweight and obesity in Kenya.

A total of 365 women aged 25–54 years old were randomly selected to participate in the study.

**Results:**

Higher age, higher socio-economic (SE) group, increased parity, greater number of rooms in the house, and increased expenditure showed greater mean body mass index (BMI),% body fat and waist circumference (WC) at highly significant levels (p <0.001). Most of the variance in BMI was explained by age, total physical activity, percentage of fat consumed, parity and SE group in that order, together accounting for 18% of the variance in BMI. The results suggest that age was the most significant predictor of all the dependent variables appearing first in all the models, while parity was a significant predictor of BMI and WC. The upper two SE groups had significantly higher mean protein (p <0.05), cholesterol (p <0.05) and alcohol (p <0.001) intakes than the lower SE groups; while the lower SE groups had significantly higher mean fibre (p <0.001) and carbohydrate (p <0.05) intakes. A fat intake greater than 100% of the DRI dietary reference intake (DRI) had a significantly greater mean BMI (p <0.05) than a fat intake less than the DRI.

**Conclusions:**

The predictors of overweight and obesity showed that urbanization and the nutrition transition were well established in the sample of women studied in the high SE groups. They exhibited a sedentary lifestyle and consumed a diet high in energy, protein, fat, cholesterol, and alcohol and lower in fibre and carbohydrate compared with those in the low SE groups.

## Background

Overweight and obesity are modifiable risk factors for the development of non-communicable diseases (NCDs). Recent evidence indicates that overweight and obesity are increasing in sub-Saharan Africa, including Kenya, at a rate of 5% per year on average [1]. Hence it is predicted that there will be an accompanying surge in NCDs such as cardiovascular diseases and type 2 diabetes. Overweight and obesity in SSA are most common in women and specifically in the 25 to 44 year old age group. This is most probably due to the retention of gestational weight gain [2]; but may also be the outcome of numerous lifestyle factors, including poor diet and physical inactivity [3]. Urbanization also plays a significant role when people are exposed to a diet which veers from that of their traditional one by containing a high intake of saturated fat, sodium, and added sugar and a low intake of dietary fibre [4].

The Kenya Demographic and Health Survey (KDHS) of 2009 showed that the national prevalence of overweight and obesity for women (15–49 years old) in Kenya was 23%. The proportion of overweight and obese women was higher in urban areas than in rural areas, with Nairobi having the highest prevalence of 41% [5].

Obesity plays a significant role in causing poor health in women, negatively affecting quality of life and shortening quantity of life [6]. There are many obesity-related conditions, which uniquely or mostly affect women. These include: osteoarthritis, birth defects, breast and endometrial cancers, cardiovascular and gall bladder diseases, infertility and gynaecological complications, urinary stress incontinence, and stigma/discrimination [7]. Women who are overweight or obese are at a higher risk of developing these conditions compared to those who are not. A direct association has been found between body weight and deaths from all-causes in women, ages 30 to 55. According to the American Obesity Association, when BMI exceeds 30 kg/m^2^, the relative risk of death related to obesity increases by 50 percent. Obesity, especially abdominal obesity, is central to the metabolic syndrome and is strongly related to polycystic ovary syndrome (PCOS) in women. Obese women are particularly susceptible to diabetes, and diabetes, in turn, puts women at dramatically increased risk of cardiovascular diseases [8].

Overweight and obesity in developing countries, has been neglected as most attention is concentrated on famine and under-nutrition or malnutrition of children [9-11]. If preventive measures are not put in place, the problem will escalate and overburden the health care system in these areas. Hence there is need to put measures in place to arrest the problem of overweight and obesity and to prevent the negative consequences, especially among women. However, in order to do this it is necessary to examine the problem and to fully understand the aetiology of obesity and the factors which give rise to excess weight gain in women. Hence the aim of the present study was to determine which factors are predictors of overweight in women in an urban setting of Kenya.

## Methods

### Study setting and participants

The study was undertaken in Kibera Division of Nairobi Province, an urban area. The Province was purposively selected because it was found to have the highest prevalence of overweight and obesity, namely 39%, in the 2003 KDHS and 41% in the KDHS of 2009 [5]. Kibera had a total adult female population of 127 656 in the most recent census. A sample of 365 adult women aged 25–54 years were required to meet sample size requirements [12]. Stratified random sampling was used according to five socio-economic strata (lower, upper lower, middle, lower upper, and upper) based on proportions of adult women living in each stratum. This resulted in four strata with 75 women and one (upper) with 36 women. One sub-location was selected at random from the division to represent each socio-economic group. All households in each sub-location were counted and households were randomly selected from these.

### Research tools

Two research assistants having a bachelor’s degree were trained to collect data on: socio-demographic status, physical activity, dietary intake and anthropometry. The main instrument of data collection was an interviewer-administered, structured questionnaire. The questionnaire was divided into four parts. The first part was used to collect socio-demographic and socio-economic status data. The second part was used to collect data on physical activity during work, transportation and leisure time in a typical week. The third part was used to collect information on dietary intake and eating habits. This was done using a food frequency questionnaire and a 24-hour dietary recall. The final part of the questionnaire was used to collect anthropometric data (weight, height, waist circumference, and body fat percentage).

#### Socio-demographic status

The socio-demographic questionnaire (SDQ) involved asking the respondents about their age, marital status and parity, while the socio-economic status questions elicited the following information: Area of residence, income status, main occupation, type of dwelling, fuel used for cooking, sources of income and monthly expenditure on selected items, water source and ownership/possession of various household items. These household items included a television, radio, refrigerator, cooker (with oven), sofa set, microwave, home computer, mobile phone, landline, land/plot, and a vehicle.

#### Physical activity

The Global Physical Activity Questionnaire (GPAQ) was used to collect data on type, frequency, duration and intensity of physical activity during work, transportation and leisure time in a typical week [13]. This is a tool that was developed by the World Health Organization (WHO) for physical activity surveillance in countries, including developing countries. The GPAQ has been validated in nine countries [13].

#### Dietary intake

In this study the food frequency questionnaire (FFQ) was used to obtain information on the type and frequency of foods consumed by the respondents in the preceding 7 days. Various foods from different food groups were read out to the respondent, who in return was required to state the number of times she had consumed this food in the preceding 7 days. Portion sizes were not calculated since only food intake and not nutrients were required.

A 24-hour recall (24 HR) was used to determine nutrient intakes and portion sizes were calculated. This method has been used in numerous studies in Africa [14-16]. One such study in Kenya used a repeated 24HR and found that there were no specific differences between the two recalls [16], hence negating the expense and time of doing a second recall. The reason for this is thought to be due to the low variety of foods eaten. Furthermore, using both the FFQ and 24 HR ensured that a check was provided for food taken in by the 24HR recall.

The 24 HR involved asking the participants to report on all foods and drinks consumed in the previous 24 hours (the previous day), in direct chronological order from the first foods in the morning to the last foods before going to bed. Probing allowed the interviewer to obtain information on forgotten foods. A range of local household utensils: glasses, spoons, cups and plates were used for estimating the amount of foods and beverages actually consumed by the respondents. The use of these local utensils acted as visual aids to increase the accuracy of portion size estimations. In some instances the respondent was asked to supply her own utensils for the recall. Seasonal fruits and vegetables were also purchased to help in estimation of the portion sizes. Rulers and tape measures were used to obtain quantities of some foods such as sugarcane and maize [17]. To obtain the weight/ gram equivalents of foods, the South African Food Photo Manual [18] was used, which converts food items of different sizes and composition to gram equivalents. In addition, the respondents were asked to provide information on the following: type of fat/oil used for cooking, preferred cooking method, meal consumption patterns in the preceding three months and the number of times food was eaten away from home.

#### Anthropometry

Anthropometric measurements of height, weight, waist circumference (WC) and percent body fat were taken to determine nutritional status [11]. Height (in metres) was measured using a steel tape which was anchored to a flat wall, and the respondent was asked to stand on a flat surface. A wooden head rest was placed on the head, which allowed the measurement to be taken at the point perpendicular to the top of the head. Weight was measured to the nearest 100 grams (0.1 kg) using a Soehnle bathroom scale, after calibrating it to zero, and after removal of shoes and excess clothing. Both weight and height were taken twice. In order to ensure quality data, the weighing scale was calibrated before measuring of weight began everyday and after every five measurements during the measuring exercise. Percentage body fat (% BF) was measured using a Soehnle scale which estimates the body fat percentage by the bioelectrical impedance analysis (BIA) method. Data on height, weight, sex and age of the participant was fed to the unit. Weight and body fat percentage values were read from the display unit of the device. Waist circumference (WC) was measured using a non-stretchable tape halfway between the lower border of ribs and the iliac crest on a horizontal plane, while ensuring that the tape was level around the body and parallel to the floor. The tape was tightened around the body without depressing the skin. Two measurements to the nearest 0.5 cm were taken and the mean recorded [11].

### Validity and reliability

Content validity of the SDQ questionnaire was tested by three experts in the field of overweight and obesity research, who were requested to assess the relevance of the content used in the questionnaire [19]. They examined the questionnaire individually and provided feedback to the researcher. Their recommendations and suggestions were integrated and incorporated in the final questionnaire.

The test re-test method [20] was used to test consistency (reliability) of the questionnaires in producing the same results. The pre-test sample comprised of 10 women who were randomly sampled outside the study area. The same questionnaire was administered to the same group of respondents after a period of one week in order to establish the extent to which the contents of the questionnaire were consistent in eliciting the same responses every time the instrument was administered. Areas of the questionnaire that were found to be deficient were revised and the questionnaire adapted accordingly.

### Pilot study

The questionnaires were pre-tested for accuracy and clarity prior to the main study on a sample of 37 women (10% of the sample size) with similar characteristics to the actual sample, but who were not included in the final study.

### Data analyses

Completed questionnaires were checked on a daily basis for accuracy and completeness in recording of responses. They were edited and coded before data entry. Before analysis, all the data were cleaned. A dietary software programme [21] was used to calculate energy and macronutrient content of the diet while SPSS was used for other data analyses. Chi-square tests were performed to establish the association between categorical variables like age group, marital status, and expenditure, while ANOVA was used to compare the means for age, income, expenditure, anthropometric measurements and the mean nutrient consumption between the five socio-economic groups. Multiple regression analysis using the stepwise method was done to determine the independent variables that were significant predictors of overweight and obesity in the study. A p value of less than 0.05 was considered to be significant.

### Ethical approval

Permission for the study was obtained from the research committee of the Ministry of Education. Ethical approval was obtained from the Kenyatta National Hospital Ethics and Research Committee. Interviews and measurements of the participants were done upon obtaining their informed and voluntary consent after the purpose of the study had been explained to them.

## Results

Results of the FFQ (Table 1) indicated that in the preceding 7 days 93% of participants had consumed sugar, 92% had whole milk, 81% had beef, 82% had kale, 80% had bananas, 77% had white rice, 76% had cabbage; 70% had sifted ugali (maize meal), 64% had dried beans, 63% had sweetened soft drinks, 61% had eggs, 57% had maize, 45% had mandazi (fried balls of bread dough). For nearly all items with exception of maize, milk, and groundnuts there were significant differences between the SE groups.

**Table 1:** Percent women consuming different foods in the preceding 7 days by socio-economic status

	**Lower**	**Upper-lower**	**Middle**	**Lower-upper**	**Upper**	**Total**	**Chi-square P value**
**Cereals/roots**							
Rice	62.7	68.9	90.5	83.3	80.6	76.9	p <0.001**
Maize porridge	76.0	63.5	83.3	75.7	30.6	70.0	p <0.001**
Maize whole	72.0	54.1	67.6	55.4	33.3	57.0	p = 0.114
Potato chips	26.5	50.0	48.6	60.8	63.9	28.2	p <0.001**
Mandazi	68.0	63.5	36.5	29.7	8.3	45.0	p <0.001**
**Dairy products**							
Whole cows milk	93.3	89.2	93.2	93.2	91.7	92.2	p = 0.659
Yogurt	9.3	18.9	28.4	52.7	66.7	31.5	p <0.001**
Ice-cream	2.7	9.5	28.4	45.9	63.9	26.1	p <0.001**
**Meat & eggs**							
Beef	70.7	74.3	83.8	93.2	83.3	80.8	p <0.001**
Fish	78.0	62.2	43.2	48.6	75.0	58.6	p <0.001**
Chicken	38.7	28.4	55.4	67.6	69.4	49.8	p <0.001**
Eggs	45.3	59.5	58.1	74.3	77.8	61.3	p <0.001**
**Legumes & nuts**							
Dry beans	58.7	60.8	82.4	67.6	33.3	63.7	p <0.001**
Groundnuts	57.3	62.2	58.1	59.5	77.8	38.7	p = 0.672
**Sugar, sweetened drinks & treats**							
Added sugar	97.3	93.2	94.6	87.7	86.1	92.5	p <0.006*
Biscuits	13.3	35.1	28.4	39.2	58.3	32.1	p <0.001**
Sweets	18.7	35.1	33.8	36.5	25.0	30.3	p = 0.007*
Sweet drinks	52.0	56.8	75.7	68.9	63.9	63.4	p = 0.006*
Alcohol	12.0	17.6	17.6	47.3	72.2	28.8	p <0.001**
Spreads (fats)							
Margarine	40.0	47.3	77.0	39.2	16.7	47.1	p <0.001**
Butter	0.4	1.4	12.2	29.7	47.2	15.0	p <0.001**
**Fruits/vegetables**							
Bananas	68.0	75.7	86.5	89.2	83.3	80.2	p <0.001**
Apples	14.7	27.0	33.8	56.8	75.0	37.5	p <0.001**
Kale	90.7	94.6	82.4	74.3	50.0	81.7	p <0.001**
Cabbage	90.7	67.6	71.6	91.9	88.9	75.7	p <0.001**

*Significant difference at p <0.01; **Significant difference at p <0.001;mandazi = deep fried doughnut.

Table 2 presents data on weight status by selected socio-demographic characteristics. With the exception of education level and occupation there is an increase in mean BMI,% BF and WC with SE characteristics. Higher age, higher SE group, increased parity, greater number of rooms in the house, and increased expenditure showed greater mean BMI,%BF and WC at highly significant levels (p <0.001). Divorced and widowed women also have greater mean BMI and WC values.

**Table 2:** Mean body mass index, percent body fat and waist circumference of women (n = 365) by selected socio-demographic characteristics

	**Mean BMI (SD)**	**Mean% body fat (SD)**	**Mean WC (SD)**
Mean	27.9 (5.3)	39.5 (7.6)	86.9 (13.5)
**Age (years)**	P <0.001^c^	P <0.001 ^c^	P <0.001 ^c^
25-29	25.6 (5.0)	35.7 (6.6)	80.7 (11.2
30-34	28.1 (4.4)	39.3 (6.3)	86.0 (11.0)
35-39	28.7 (4.6)	40.8 (6.7)	87.7 (8.7)
40-44	28.9 (5.5)	41.7 (7.5)	90.1 (9.1)
45-49	28.8 (6.5)	41.2 (7.8)	90.9 (18.4)
50-54	31.8 (4.7)	46.6 (7.8)	99.5 (14.6)
**Socio-economic group**	P = 0.005^b^	P = 0.021^a^	P = 0.083
Lower	26.4 (5.2)	37.6 (6.8)	86.1 (12.2)
Upper-lower	27.6 (4.8)	39.2(7.9)	85.9 (12.2)
Middle	27.7 (4.9)	39.3(7.8)	84.5 (11.8)
Lower-upper	29.5 (%.3)	41.1(7.1)	90.3 (14.7)
Upper	29.1 (6.3)	42.1 (8.3)	88.8 (17.7)
**Marital status**	P = 0.01^a^	P = 0.06	P <0.001 ^c^
Single	26.1 (4.8)	37.8 (8.0)	82.4 (11.9)
Married	28.3 (5.4)	39.9 (7.1)	87.6 (13.2)
Divorced/separated	30.1 (4.8)	42.5 (8.9)	93.0 (16.2)
Widowed	29.5 (4.7)	41.1 (8.5)	93.0 (14.3)
**Parity**	P <0.001 ^c^	P <0.001 ^c^	P <0.001 ^c^
0-1 child	26.4 (5.0)	37.6 (7.3)	82.5 (12.8)
2-3children	28.1 (4.6)	39.8 (6.6)	86.5 (11.5)
> = 4 children	30.0 (5.8)	42.1 (8.8)	94.3 (14.2)
**Education level**	P = 0.744	P = 0.764	P = 0.123
None	27.7 (5.3)	40.1 (8.9)	89.1 (15.2)
Primary	28.4 (5.4)	39.9 (7.3)	89.0 (13.1)
Secondary	27.6 (6.4)	38.7 (8.8)	85.8 (14.9)
>Secondary	27.8 (4.7)	39.5 (6.9)	85.3 (12.4)
**Occupation**	P = 0.366	P = 0.471	P = 0.314
Casual worker	26.9 (6.3)	37.4 (7.9)	86.6 (15.7)
Self employed	28.7 (5.3)	40.3 (7.5)	89.2 (13.5)
Formal sector	27.7 (4.8)	39.4 (6.9)	85.5 (12.3)
Housewife	27.6 (5.5)	39.7 (8.4)	86.1 (13.7)
Other	26.9(6.0)	38.0 (10.1)	86.0 (17.3)
**Number rooms**	P <0.001 ^c^	P <.0.001 ^c^	P <0.001 ^c^
1-2	26.8 (5.4)	37.6 (7.0)	84.9 (12.4)
3-4	27.8 (4.6)	40.0 (7.0)	85.4 (11.5)
5-6	30.3 (5.4)	42.7 (7.6)	92.6 (15.4)
> = 7	28.9 (5.2)	40.9 (8.5)	90.7 (16.2)
**Expenditure (Ksh)**	P <0.001 ^c^	P <0.001 ^c^	P = 0.014^a^
<10 000	26.2 (5.1)	37.2 (7.7)	83.8 (12.7)
10 001–20 000	28.1 (5.3)	39.8 (7.2)	86.8 (12.5)
20 001–30 000	28.9 (4.8)	41.4 (7.5)	88.8 (12.4)
30 001–40 000	29.4 (5.6)	40.4 (8.1)	91.7 (16.7)
> = 40 000	29.7 (4.9)	42.3 (6.7)	89.4 (14.7)

BMI = body mass index; WC = waist circumference; SD = standard deviation; Ksh = Kenyan shillings.

^a^ Significant differences at p <0.05; ^b^ Significant differences at p <0.01; ^c^ Significant differences at p <0.001.

The largest percentage women with high physical activity levels (PALs) are found in the lowest three SE groups (p <0.001) (Table 3). These three groups also have the lowest levels of sedentary time (p <0.0001). There is a significant increase in mean BMI (p <0.001),% BF (p <0.01) and WC (p <0.05) as PAL levels decrease with the highest mean BMI,%BF and WC found at the lowest level of PAL (Table 4). There is a similar trend with increasing sedentary time which is only significant for BMI; with the highest mean BMI having the highest level of sedentary time (p <0.05).

**Table 3:** Percentage time spent on physical activity by the Kenyan women according to socio-economic group

	**Lower**	**Upper-lower**	**Middle**	**Lower-upper**	**Upper**	**Total**	**Chi-square**
**PAL**	**N (%)**	**N (%)**	**N (%)**	**N (%)**	**N (%)**	**N (%)**	
High	61 (32)	44 (23)	45 (23)	28 (15)	14 (7)	192 (58)	P <0.001^a^
Moderate	7 (10)	17 (28)	13 (19)	16 (24)	12 (18)	67 (20)	
Low	7 (10)	11 (15)	16 (22)	29 (40)	10 (14)	73 (22)	
**Sedentary time per day**							
< 5 hours	31 (37)	19 (22)	16 (19)	13 (15)	6 (7)	85 (26)	P <0.001^a^
5-10 hours	23 (18)	35 (27)	33 (25)	20 (15)	19 (15)	130 (39)	
>10 hours	21 (18)	20 (17)	25 (21)	40 (34)	11 (9)	117 (35)	

PAL = physical activity level; ^a^ Significant differences at p <0.001.

**Table 4:** Mean body mass index, percent body fat and waist circumference of women by physical activity levels

	**Mean BMI (SD)**	**Mean% body fat (SD)**	**Mean WC (SD)**
Mean	27.9 (5.3)	39.5 (7.6)	86.9 (13.5)
**PAL**	P <0.001^c^	P = 0.004^b^	0.040^a^
High	27.0 (5.1)	38.6 (7.1)	85.4 (12.9)
Moderate	28.4 (5.6)	39.5 (7.3)	88.6 (13.8)
Low	29.9 (4.9)	42.1 (8.5)	89.6 (14.1)
**Sedentary time**	P = 0.014^a^	P = 0.126	P = 0.137
< 5 hours/day	27.2 (5.4)	39.5 (7.5)	86.5 (13.7)
5-10	27.7 (5.1)	38.8 (7.5)	85.9 (13.2)
>10	29.4 (5.1)	41.1 (7.9)	89.7 (13.4)

BMI = body mass index; WC = waist circumference; SD = standard deviation; PAL = physical activity level.

^a^ Significant differences at p <0.05; ^b^ Significant differences at p <0.01; ^c^ Significant differences at p <0.001.

Table 5 shows that the upper two SE groups have significantly higher mean protein (p <0.05), cholesterol (p <0.05) and alcohol (p <0.001) intakes than the lower SE groups; while the lower SE groups have significantly higher mean fibre (p <0.001) and carbohydrate (p <0.05) intakes. This was due to a significantly more frequent consumption of beef, chicken, processed meats and eggs by the highest two SE groups; while the lowest two groups consumed significantly more dry beans and maize meal (not shown). Both BMI (p <0.05) and% BF (p <01) are significantly greater when protein intake is higher than 100% of the DRI (Table 6). This is also the case for mean fat intake. A fat intake greater than 100% of the DRI has a significantly greater mean BMI (p <0.05) than a fat intake less than 100% of the DRI.

**Table 5:** Comparison of energy and macronutrient intakes as a percentage of Dietary Reference Intakes of women in different socioeconomic groups

	**Lower**	**Upper-lower**	**Middle**	**Lower-upper**	**Upper**	**Total**	**Chi-square p values**
Energy% DRI	115.3^a^	116.5 ^a^	113.2^a^	129.5 ^a^	113.9 ^a^	117.9	0.430
	50.1	44.3	56.9	63.1	66.7	55.4	
Protein% DRI	160.3	153.7 ^a^	177.6 ^a^	206.7	208.9	177.8	0.034^d^
SD	99.8	66.3	94.4	104.6	259.3	123.2	
Fat% DRI	73.1 ^a^	80.9 ^a^	89.2 ^a^	93.8 ^a^	77.2 ^a^	83.1	0.13
SD	37.5	65.7	49.3	53.2	46.4	52.0	
CHO% DRI	99.8 ^a^	101.5 ^a^	91.3 ^a^	88.0 ^a^	71.4	92.7	0.016 ^d^
SD	44.3	41.2	56.9	44.1	48.9	47.9	
Fibre% DRI	150.7 ^a^	146.3 ^a^	116.1 ^a^	104.2	82.5	124.6	<0.001^f^
SD	74.1	76.8	115.2	103.2	76.9	94.5	
PUFA% DRI	137.4 ^a^	166.0 ^a^	179.5	171.8	123.5 ^a^	159.1	0.078
SD	102.6	119.3	112.9	159.3	94.5	122.5	
Cholesterol DRI	152.2 ^a^	147.9 ^a^	185.2	219.4	97.1 ^a^	166.7	0.011^d^
SD	166.9	146.0	185.0	223.6	165.9	182.7	
Alcohol mean (g) SD	1.8 ^a^	0.7 ^a^	1.1 ^a^	45.3	78.2	18.8	<0.001^f^
	11.9	6.2	9.2	127.2	239.8	101.6	

DRI = Dietary Reference Intakes SD = standard deviation; CHO = carbohydrate.

^a,b,c^ Significant differences between means if letter is not the same (Bonferroni test); ^d^ Significant difference p <0.05; ^f^ Significant difference p <0.001.

**Table 6:** Mean body mass index, percent body fat and waist circumference of women by energy and macronutrient levels

	**Mean BMI (SD)**	**Mean% body fat (SD)**	**Mean WC (SD)**
Mean (SD)	27.9 (5.3)	39.5 (7.6)	86.9 (13.5)
Energy (mean DRI)	P = 0.301	P = 0.717	P = 0.775
<100%	27.6 (5.3)	39.4 (8.0)	86.6 (14.6)
>100%	28.2 (5.2)	39.7(7.2)	87.1 (12.7)
Protein (mean DRI)	P = 0.013^a^	P = 0.004 ^b^	P = 0.064
<100%	26.5 (5.1)	37.2 (8.3)	84.0 (13.9)
>100%	28.2 (5.1)	40.1 (7.3)	87.0 (12.9)
Fat (mean DRI)	P = 0.012 ^a^	P = 0.174	P = 0.201
<100%	27.5 (5.2)	39.2 (7.8)	86.3 (13.5)
>100%	29.1 (5.3)	40.5 (7.3)	88.4 (13.7)
CHO (mean DRI)	P = 0.232	P = 0.309	P = 0.695
<100%	27.7 (5.4)	39.2 (7.9)	86.6 (14.4)
>100%	28.4 (5.1)	40.1 (7.1)	87.3 (11.7)

BMI = body mass index; WC = waist circumference; SD = standard deviation; CHO = carbohydrate.

^a^ Significant differences at p <0.05; ^b^ Significant differences at p <0.01.

Most of the variance in BMI was explained by age, total physical activity, percentage DRI of fat consumed, parity and socio-economic group, in that order, together accounting for 18.0% of the variance in BMI (Table 7). The most significant predictors of overweight and obesity by fat percentage were age and the number of items/assets in the women’s households, both of which explained 17.8% of the variation in the body fat percentage of the women. With regard to WC, age, parity and the number of rooms in the houses where the women resided, were the most significant predictors of abdominal fat deposition. These three variables together accounted for 20.6% of the variation in the WC measurements. Significant variables are shown in Table 7. The results suggest that age was the most significant predictor of all the dependent variables appearing first in all the models, while parity was a significant predictor of BMI and WC.

**Table 7:** Multiple linear regression showing the effects of selected variables on body mass index, percent body fat and waist circumference of women using the stepwise method

	**BMI**		**% Body fat**		**Waist circumference**	
**Predictor variables**	**β**	**P**	**β**	**P**	**β**	**P**
Age	0.177	0.008^a^	0.378	<0.001 ^a^	0.237	<0.001 ^a^
% DRI fat	−0.122	0.024 ^a^	−0.021	0.689	−0.040	0.447
Total PA	0.115	0.026 ^a^	0.093	0.071	0.075	0.132
Parity	0.209	0.002^a^	0.081	0.211	0.237	<0.001 ^a^
SE group	0.144	0.015 ^a^	0.004	0.966	−0.036	0.660
No. of assets	0.055	0.534	0.139	0.007**	0.007 ^a^	0.922
No. rooms	0.031	0.710	0.032	0.666	0.125	0.022 ^a^
R^2^						

BMI = body mass index; WC = waist circumference; SE = socio-economic; DRI = Dietary Reference Intakes; TPA = total physical activity; ^a^ Significant predictors of BMI,% body fat, and waist circumference; β Standardised beta coefficient gives a measure of the contribution of each variable to the model.

## Discussion

The study demonstrated a significantly higher prevalence of overweight and obesity among women as the socio-economic status (determined by residential area, income and expenditure, number of household assets, number of rooms in the house), age and parity increased; while physical activity was seen to have a protective effect on BMI and abdominal obesity. This high prevalence of overweight and obesity is a clear indication that the study population, and probably women in other urban areas of SSA, may be vulnerable to co-morbidities associated with being overweight or obese. These results are consistent with the findings of earlier studies in SSA countries [22,23].

The finding that the BMI of Kenyan women followed a socio-economic gradient; being lowest in the lower SE category and highest in upper income women is an interesting feature since it is in direct contrast to the USA where the socio-economic gradient is reversed and lower income women have the highest rates of overweight and obesity [24]. Studies in the USA have shown a significant association between obesity and food insecurity in women, particularly in minority groups [25,26].

These results are comparable to an earlier study in Kenya which demonstrated that urban women in the higher SE groups had a higher prevalence of obesity as illustrated by both BMI and WC [16]. This is postulated to be due to urbanization with a resulting westernization of diet as more convenience and fast foods become available. While these foods may be affordable to the higher SE group it is likely that they may still be out of the price range of the lower income group. This was also clearly illustrated in this study by the finding that women in the higher income groups ate more meals away from home. It was interesting to note that there appeared to be no significant differences in BMI, WC and percent body fat by level of education in the present study. This is different to an earlier study [16] in women in Kenya where overweight was found to be highest in the higher education group. The most likely cause for this is the fact that the earlier study comprised a representative sample from urban and rural areas while the current study was done in an urban area known to have a high prevalence of obesity. It is known that the prevalence of obesity is generally higher in urban areas of African countries [27].

The dietary intake of the women in the present study confirms what we know about the nutrition transition [28]. Those women having the highest protein (beef, chicken, processed meats) and fat intake also had the highest mean BMI. Furthermore, those in the highest income group were the most sedentary and fewer women had high levels of physical activity. This is also supported by the predictors of overweight since most of the variance in BMI was explained by age, total physical activity, amount of fat consumed, parity and SE group; the classic picture of urbanization and westernization of lifestyle [29,30].

The low levels of physical activity (especially in the upper SE groups) in this study could be an indication that this could be the situation in other geographical locations and population groups. Hence there is a need for concerted effort to promote increased physical activity. Interventions on lifestyle changes should especially target the people in the upper SE groups, and those who are better off in terms of SE status. This is justified by the rising levels of overweight and obesity up the SE ladder, and the low levels of physical activity relative to the other SE groups.

The high prevalence of overweight and obesity in all three indicators calls for public health interventions to reduce the prevalence of overweight and obesity among urban women in Kenya . Awareness programs about the consequences of overall and abdominal obesity including prevention activities should be made available in the workplace and in the community to act as reference points for the women in these areas. Even as the government of Kenya, non-governmental organizations and other stakeholders grapple with the problem of hunger and food insecurity, some effort should be directed towards overweight and obesity because of the associated health risks. This problem needs to be emphasized as the prevalence of obesity keeps increasing, and may continue to worsen unless appropriate preventive measures are taken.

## Conclusions

The predictors of overweight and obesity showed that urbanization and the nutrition transition were well established in the sample of women studied in the high SE groups. They exhibited a sedentary lifestyle and consumed a diet high in energy, protein, fat, cholesterol, and alcohol and lower in fibre and carbohydrate compared with those in the low SE groups.

### Recommendations

In terms of practice, the high rates of overweight and obesity among the women point to a need for behaviour change related to improved lifestyle through increased physical activity and improved dietary practices. This could be through increasing their physical activity, both planned and unplanned, for instance by walking or enrolling in facilities that offer planned physical activity. For women who are in lower socio-economic groups, and who cannot afford these facilities, walking or jogging could be recommended. In terms of policy, the study found that overweight and obesity is not a problem only limited to the high socio-economic groups; hence affordable interventions targeting all socio-economic groups should be put in place.

With regard to further research, studies to determine nutrition knowledge and attitudes of Kenyan women and the determinants of different behaviours that may lead to overweight and obesity (e.g. why people make the food choices they do; and perceived barriers to healthy eating and engaging in physical activity) should be done in order to understand the problem and to develop relevant, practical solutions.

## Competing interests

The authors declare that they have no competing interest.

## Authors’ contributions

EK, JK and SO were involved in planning of the study and design. RM was involved in planning, field work, data analyses and writing. NPS was involved conceptually and in writing the manuscript. All authors read and approved the final manuscript.

## Pre-publication history

The pre-publication history for this paper can be accessed here:

http://www.biomedcentral.com/1471-2458/12/823/prepub
